# Palliative care for homeless people: a systematic review of the concerns, care needs and preferences, and the barriers and facilitators for providing palliative care

**DOI:** 10.1186/s12904-018-0320-6

**Published:** 2018-04-24

**Authors:** Hanna T. Klop, Anke J.E. de Veer, Sophie I. van Dongen, Anneke L. Francke, Judith A.C. Rietjens, Bregje D. Onwuteaka-Philipsen

**Affiliations:** 1 Amsterdam Public Health Research Institute (APH), Department of Public and Occupational Health, Expertise Centre for Palliative Care, VU University Medical Center, P.O. Box 7057, 1007 MB Amsterdam, The Netherlands; 2 Netherlands Institute for Health Services Research (NIVEL), P.O. Box 1568, 3500 BN Utrecht, Netherlands; 3 Department of Public Health, Erasmus University Medical Center, P.O. Box 2040, 3000 CA Rotterdam, Netherlands

**Keywords:** Palliative care, End-of-life, Homeless people, Systematic review, Concerns, Needs, Barriers, Facilitators

## Abstract

**Background:**

Homeless people often suffer from complex and chronic comorbidities, have high rates of morbidity and die at much younger ages than the general population. Due to a complex combination of physical, psychosocial and addiction problems at the end of life, they often have limited access to palliative care. Both the homeless and healthcare providers experience a lot of barriers. Therefore, providing palliative care that fits the needs and concerns of the homeless is a challenge to healthcare providers. This systematic review aims to summarize evidence about the concerns, palliative care needs and preferences of homeless people, as well as barriers and facilitators for delivering high quality palliative care.

**Methods:**

PubMed, Embase, PsycINFO, CINAHL and Web of Science were searched up to 10 May 2016. Included were studies about homeless people with a short life expectancy, their palliative care needs and the palliative care provided, that were conducted in Western countries. Data were independently extracted by two researchers using a predefined extraction form. Quality was assessed using a Critical Appraisal instrument. The systematic literature review was based on the PRISMA statement.

**Results:**

Twenty-seven publications from 23 different studies met the inclusion criteria; 15 studies were qualitative and eight were quantitative. Concerns of the homeless often related to end-of-life care not being a priority, drug dependence hindering adequate care, limited insight into their condition and little support from family and relatives. Barriers and facilitators often concerned the attitude of healthcare professionals towards homeless people. A respectful approach and respect for dignity proved to be important in good quality palliative care.

**Conclusions:**

A patient-centred, flexible and low-threshold approach embodying awareness of the concerns of homeless people is needed so that appropriate palliative care can be provided timely. Training, education and experience of professionals can help to accomplish this.

**Electronic supplementary material:**

The online version of this article (10.1186/s12904-018-0320-6) contains supplementary material, which is available to authorized users.

## Background

Homeless people are those without permanent housing, e.g. living in sheltered housing or on the streets [1, 2]. It is known that homeless people often have substance abuse problems, high rates of mental illness and serious physical illness, lack of social support, and lack of health insurance [3–8]. Many of them suffer from complex and often chronic comorbidities, such as liver cirrhosis, cancer and HIV [6, 9, 10]. In addition, they die at much younger ages than the general population [7, 11–14].

It is therefore evident that a large proportion of homeless people can benefit from palliative care. According to the widely accepted definition of the World Health Organization (WHO), “palliative care is an approach that improves the quality of life of patients and their families facing the problems associated with life-threatening illness, through the prevention and relief of suffering by means of early identification and impeccable assessment and treatment of pain and other problems, physical, psychosocial and spiritual” [15]. The definition shows that palliative care covers a broad range of domains and can start in an early phase of a life-threatening illness. Given the multiple problems homeless people have, it is apparent that providing good and accessible palliative care to homeless people a challenge.

Until now, research conducted on this topic has been addressed in three reviews [10, 16, 17]. First, Sumalinog et al. reviewed the effectiveness of three interventions during homeless people’s final stage of life, including: an intervention encouraging the completion of advance directives, a shelter-based palliative care programme, and an intervention aiming to improve cooperation between palliative care services and social services for the homeless. They tentatively conclude that there is some evidence that the interventions lead to the completion of more advance care directives and better access to palliative care [10]. In addition, a review by Hubbell also focused on the completion of advance care planning, concluding that clinician-guided interventions with homeless individuals were effective in getting advance directives completed and in obtaining surrogate decision-makers. Hubbell also found that homeless people had several concerns at the end of life, such as a fear of dying alone and concerns regarding burial and notification of family [17]. Furthermore, Hudson et al. summarized the findings in qualitative studies on palliative care among homeless people to get a better understanding of the challenges for palliative care access and delivery [16]. In the review by Hudson et al., three types of challenges were identified, which they described as challenges related to chaotic lifestyles, challenges concerning the delivery of end-of-life care in hostels, and the challenges of caring for homeless people in mainstream palliative care settings.

While the three reviews provide valuable information, they do not provide a complete overview of the existing literature on palliative care for homeless people. First of all, the reviews of Sumalinog et al. and Hubbell focus exclusively on the terminal phase of life, excluding earlier stages of the palliative care trajectory. Additionally, both reviews of Sumalinog et al. and Hubbell are mainly concerned with structure (such as cooperation), ethical decisions (such as advance directives) and homeless people’s attitudes towards dying. These two reviews do not look at the care needs of homeless people and how to meet these needs. Furthermore, Hudson’s review limits itself to qualitative studies and only focuses on challenges concerning the access and delivery of palliative care, without looking at possibilities for improvements. Given the relatively narrow focus of each of the three previous reviews, we found the need for a more comprehensive review providing a broader overview of relevant literature on palliative care for homeless people. In this review we offer such a comprehensive overview by using the broad definition of palliative care as defined by the WHO, which emphasizes care in four domains - somatic, psychological, social and spiritual - and also recognizes that care can start before the terminal phase. Besides this, by looking at the possibilities available for providing good palliative care (barriers and facilitators), and by including both qualitative, quantitative and mixed-method studies, this review contributes to the existing literature.

In order to provide palliative care tailored to the needs of homeless people, the objective of this systematic review is to summarize what evidence already exists about concerns and healthcare needs, as well as the conditions for delivering good quality palliative care for the target group. The research questions are therefore:

1. What is known from previous research about the concerns, care needs and preferences of homeless people regarding palliative care?
2. What is known from previous research about what barriers and facilitators are found in the delivery of palliative care for homeless people?
3. What is known from previous research about recommendations for practice regarding palliative care to homeless people?

## Methods

### Design and eligibility criteria

A systematic review of the research literature was carried out to identify studies that examined the concerns and needs in palliative care for homeless people, and/or provided care to seriously ill homeless people. A review protocol was developed based on the Preferred Reporting Items for Systematic Reviews and Meta-Analysis (PRISMA) statement [18].

Studies eligible for inclusion had to meet the following criteria:

1. The study concerns homeless people who provided information about their views, wishes, and/or preferences towards the end of life, including homeless people having a life limiting condition.
2. The study includes data derived from homeless people themselves, from their healthcare professionals or data from registration, medical files or cohorts (either qualitative or quantitative).
3. The study concerns the palliative care provided (somatic, psychological, social and/or spiritual), factors influencing that care, palliative care needs and/or care interventions or innovations for palliative care.

Commentaries, editorials, abstracts, posters for conferences and non-empirical studies were excluded. In addition, studies conducted outside the Western World (outside Northern, Eastern, Southern and Western Europe or Anglo Saxon countries) were excluded. Since Western countries already differ in the way care for homeless people is organized within the health and welfare system, we did however want to ensure comparability in terms of living conditions and welfare levels. There were no restrictions on the setting, year of publication and language of the publication.

### Searches

**Fig. 1:**
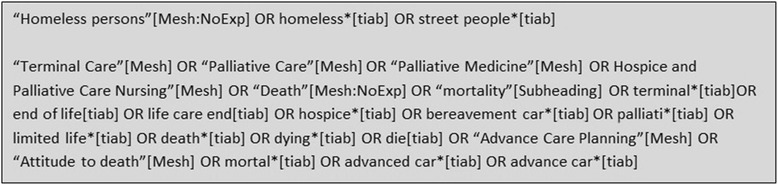
Search string PubMed

The following sources were searched from inception: Embase.com and Ebsco/PsycInfo (up to 1 April 2016), Ebsco/CINAHL (up to 5 April 2016), Thomson Reuters/Web of Science (up to 3 May 2016) and PubMed (up to 10 May 2016). To identify studies about homelessness and palliative care, we used a pre-defined search strategy. The string for PubMed is shown in Fig. 1, detailed information for all search strings is shown in Additional file 1.

References listed in review articles and references in papers which were excluded in the full text round were also checked. In order to find grey literature, relevant websites of organizations that are involved in palliative care for homeless people or research into it were consulted by searching for relevant keywords using Google (e.g. Simon Communities Ireland – Homeless Charity and St. Mungo Community Housing Association). Duplicate articles were excluded.

### Study identification and data extraction

All the references obtained by searching databases as mentioned above were independently reviewed by two researchers, using Covidence online software (a primary screening and data extraction tool) [19]. Firstly, titles and abstracts were screened in order to determine whether studies met the eligibility criteria. The exclusion criteria were (1) homeless people could not be distinguished as a separate subgroup (2) study was not about somatically ill homeless adults with a short-life expectancy (3) search outcomes included: comments, editorials, abstracts and posters (4) study was not conducted in N-E-W Europe or Anglo-Saxon countries, and (5) study was not about palliative/end-of-life care. Cohen’s kappa for the first selection of titles and abstracts was 0.92 (unweighted), which is almost perfect according to Landis & Koch [20]. In the second round, the remaining full text papers were independently assessed by two reviewers against inclusion criteria. Cohen’s kappa for the second round was 0.81 (unweighted), thus also reflecting almost perfect agreement according to Landis & Koch [20]. Disagreements about whether or not the criteria were met were solved by discussion and a third researcher was consulted in the event of disagreement. There was disagreement in 8 of the 91 studies (8.8%).

For data extraction and analyses we followed the assumptions for an integrated design of a systematic review, which indicate that qualitative, quantitative and mixed-method studies can be jointly analysed and synthesized [21]. The extraction form was developed by two researchers, discussed by the research group and adjusted in response to comments. Extracted data included information about the country of the research, the research aims and questions, methods and data collection, characteristics of participants, setting, perspective of the publication (homeless people, healthcare providers, relatives/friends, open answer questionnaire), results, strengths and limitations of the study design and key conclusions. The results were extracted with a focus on the research questions; with regard to recommendations for practice we limited ourselves to recommendations given by the authors that were related to the results found in that study. For the first five publications, two researchers extracted the data independently, without any extraction software. When necessary, adjustments were made and conflicts were resolved. For the other papers, data was extracted by one reviewer and checked by a second.

### Analysis

Because our aims were ‘to summarize evidence about the concerns, palliative care needs and preferences of homeless people, as well as barriers and facilitators for delivering high quality palliative care’, we used the findings from the selected studies mainly to describe common themes. Thus, data was analysed using the meta-summary method [21] to identify common themes. The extracted data was classified manually into categories by sorting according to common themes, carried out by one researcher until no new categories came up. These themes were then discussed with a second researcher before discussion in the project team. In the tables the common themes are shown, indicating in which studies they occurred.

### Critical appraisal of the methodological quality

The methodological quality of the studies that met the inclusion criteria was assessed the General Appraisal instrument of Hawker et al. [22]. The instrument, which is applicable to quantitative as well as qualitative studies, consists of nine elements (abstract, background, methodology, sampling, data analysis, ethics, results, transferability and implications). Each element is scored on a four-point scale (ranging from very poor to good). Scores for the various are added to give a total score. Total scores range from 9 to 36; scores less than or equal to 18 are rated as ‘poor methodological quality’, scores from 19 to 27 as ‘moderate’ and above 27 as ‘good quality’. All methodological assessments were done by two reviewers independently. If there was a mismatch of more than five points, disagreements were solved by discussion. The scores of assessment can be found in Table 1, more details of the assessments can be found in Additional file 2.

**Table 1:** Characteristics of the papers included

Reference	Aim	Country	Setting	Method	Participants	N	Informa-tion on 1,2, 3,^b^	Critical appraisal score^c^
[31]	To know more about the content of advance directives completed by homeless people who participated in a guided intervention arm	USA	Homeless drop-in shelter	Qualitative analysis of participants’ responses to individual items in an advance directive^a^	Homeless people with a terminal illness	Homeless people (*n* = 17)	1, 2, 3	24/20 Moderate
[32]	To identify the observed changes in general condition or behaviour of homeless people with advanced liver disease who may be in deteriorating health and approaching the end of life in order to better recognize an increased likelihood of death and to explore staff’s experiences of death of residents	UK	Homeless shelter	Case note review, focus groups (qualitative)	Case notes about homeless people with advanced liver disease, staff members of a supporting home for homeless people	Case notes (*n* = 27) staff members (*n* = 13)	1, 2, 3	22/21 Moderate
[37]	To explore the staff members’ experiences of and reasoning about the palliative care they provided	Sweden	Support home for homeless	Paired and individual conversations (qualitative)	Staff members of a support and housing home for homeless	Staff members (*n* = 12)	1, 2, 3	34/31 Good
[49]	To describe challenges of caring for homeless veterans at end of life as perceived by Veterans Affairs Medical Centre (VAMC) homeless and EOL care staff	USA	Veterans Affairs Medical Centres (VAMC) with programmes for homeless veterans with a short life expectancy	E-mail survey (quantitative)	Care staff of homeless and EOL programmes	50 VAMCs	2	28/23 Moderate
[43]	To assess the extent to which homeless persons may underuse healthcare services even when they are at a high risk of death and to examine potential opportunities for intervention in this population	USA	Boston Health Care for the Homeless Program	Review of medical records (quantitative)	Deceased homeless adults	Medical records (*n* = 558)	2, 3	27/25 Moderate
[23]	To explore the views, concerns, and needs regarding advance care planning among older homeless adults	USA	Transitional housing facility	Semi-structured face-to-face interviews (qualitative)	Homeless adults aged 60 and older	Homeless (*n* = 21)	1, 2, 3	30/29 Good
[24]	To explore older homeless adults’ perspectives toward good and bad deaths and their concerns regarding their EOL care needs	USA	Transitional housing facility	Semi-structured face-to-face interviews (qualitative)	Homeless adults aged 60 and older	Homeless (*n* = 21)	1, 2, 3	30/33 Good
[36]	To explore how access to Toronto’s palliative services can be improved to better serve the city’s homeless	Canada	Providers of care for the homeless	Semi-structured interviews (qualitative)	Homeless care providers with extensive experience and experience dealing with death and dying	Registered nurses (*n* = 3) outreach workers (*n* = 4)	1, 2, 3	19/18 Poor
[41]	To determine the rate of advance directive completion using a one-on-one counsellor-guided intervention	Canada	Shelter for homeless men	Counsellor-guided intervention (quantitative)^a^	Chronically homeless individuals in a managed alcohol harm reduction program^a^	Shelter residents (*n* = 205)	1, 2	31/33 Good
[47]	To identify best practice for managing the palliative care needs of clients experiencing homelessness in a community setting and to guide the development of policies for a community-based palliative care service working with these clients	Australia	Community-based palliative care service	Semi-structured individual interviews (qualitative)	Workers from hospital and community organizations	Staff members (*n* = 6)	2, 3	27/24 Moderate
[30]	To explore and describe aspects of social networks that have a potential for caregiving during the terminal phase of a disease	USA	Patients of two medical centres, living in single room buildings	Semi-structured individual interviews (qualitative)	Homeless who had been diagnosed with unresectable lung cancer	Homeless (*n* = 8)	2	21/19 Moderate
[26]	To identify challenges health and social service providers face in facilitating and delivering end-of-life care services to homeless illicit drug users	Canada	Health and social care services	Semi-structured individual interviews (qualitative)	Health and social services professionals involved in end-of-life care services delivery to homeless persons	Healthcare professionals and managers (*n* = 50)	2, 3	29/31 Good
[27]	To identify barriers to the end-of-life care system for homeless populations and generate recommendations to improve their access to end-of-life care	Canada	Health and social services	Semi-structured individual interviews (qualitative)	Health and social services professionals involved in end-of-life care services delivery to homeless persons	Healthcare professionals and managers (*n* = 54)	2, 3	32/35 Good
[25]	To explore the role of harm reduction services in end-of-life care services delivery to homeless and marginally housed persons with problematic use of alcohol and/or illicit drugs	Canada	Health and social services	Semi-structured individual interviews (qualitative)	Health and social services professionals involved in end-of-life care services delivery to homeless persons	Healthcare professionals and managers (*n* = 54)	2, 3	32/33 Good
[45]	To determine the benefits and barriers of in-shelter palliative care and possible enablers to future implementation in Toronto	Canada	Three shelters	Semi-structured individual interviews (qualitative)	Shelter-based social service providers	Case workers, social support workers, shelter managers (*n* = 5)	2, 3	23/19 Moderate
[40]	To examine the treatment preferences of homeless (in comparison with preferences of physicians likely to be providing care for homeless persons and patients with oxygen-dependent COPD)	USA	Homeless shelters, hospitals	Cross-sectional survey (quantitative)	Visitors of homeless shelters, physicians providing care to homeless persons, patients with COPD	Homeless (*n* = 229), physicians (*n* = 236), COPD-patients (*n* = 111)	1, 3	31/32 Good
[44]	To improve the understanding of elderly homeless persons and to describe the living circumstances of the group, especially housing	USA	Multidisciplinary Street Team of Boston	Analysis of an intervention^a^ (quantitative)	Elderly homeless individuals (> 50)	Homeless (*n* = 30)	2, 3	15/13 Poor
[48]	To explore if effective shelter-based palliative care could be provided to terminally ill homeless individuals at substantial cost savings	Canada	Shelter-based palliative care hospice	Analysis of records of a cohort and a five-member panel (quantitative)	Terminally ill homeless	Records of homeless (*n* = 28)	2, 3	32/30 Good
[38]	To explore the importance of end-of-life care for homeless people and the type of concerns	USA	Sites for homeless in Minnesota	Focus groups (qualitative)	Homeless individuals	Homeless (*n* = 57)	1, 2, 3	18 Poor
[33]	To understand the viewpoints of people who are homeless regarding end-of-life issues, to elucidate the barriers to good end-of-life care, and to offer insight into the most basic needs and wishes.	USA	Homeless shelter, two service organizations for homeless	Focus groups (qualitative)	Homeless individuals and social workers	Homeless (*n* = 11) service providers (*n* = 9)	1, 2, 3	23/24 Moderate
[29]	To explore the experiences and attitudes toward death and dying among homeless persons.	USA	Social service agencies which serve homeless	Focus groups (qualitative)	Homeless individuals	Homeless (*n* = 53)	1, 2, 3	30/35 Good
[28]	To examine how homelessness influences concerns and desires about care at the time of death.	USA	Social service agencies which serve homeless people	Focus groups (qualitative)	Homeless individuals	Homeless (*n* = 53)	1, 2, 3	29/32 Good
[46]	To improve the EOL decision-making process for homeless persons by facilitating ACP	USA	Drop-in centre	RCT comparing two types of interventions^a^ (quantitative)	Homeless individuals	Homeless (*n* = 59)	2, 3	32/35 Good
[42]	To determine whether homeless persons will complete a counselling session on advance care planning and fill out a legal advance directive designed to assess care preferences and preserve the dignity of marginalized persons	USA	Sites serving homeless persons	RCT comparing two type of interventions^a^ (quantitative)	Homeless individuals	Homeless (*n* = 262)	1, 2, 3	31/35 Good
[39]	To increase healthcare providers’ understanding and insight into how to better provide EOL care for homeless people.	USA	Free urban healthcare clinic for homeless individuals	Focus groups (qualitative)	Homeless individuals	Homeless (*n* = 20)	1, 2, 3	31/28 Good
[34]	To identify and examine the needs of older people who are homeless or who have previously experienced homelessness as they age and are faced with the issues of serious ill health, dying and death.	Ireland	Community where care, accommodation and support are being provided for people experiencing homelessness and those at risk	Interviews (qualitative)	Homeless individuals	Homeless (*n* = 16)	1, 2, 3	22 Moderate
[35]	To explore the views of hostel staff regarding palliative and end-of-life care for the homeless population	UK	Intermediate or long stay hostels	Semi-structured individual interviews (qualitative)	Hostel workers	Hostel workers (*n* = 7)	1, 2, 3	28/33 Good

^a^Method includes an intervention

^b^1 = Concerns, care needs and future preferences for care and treatment of seriously ill homeless people needs; 2 = the care provided: barriers and facilitators; 3 = recommendations for practice

^c^Score 1 = HTK, score 2 = AJEV

## Results

### Review selection

**Fig. 2:**
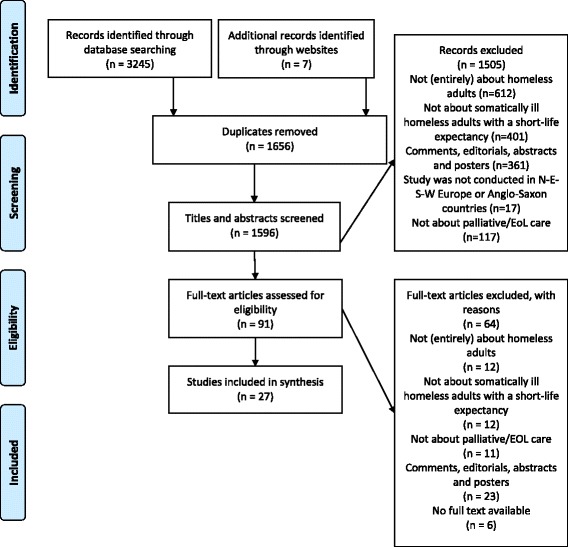
PRISMA flow diagram

The review process is shown in Fig. 2. We identified 3245 records through database searches, seven additional records were found through websites of organizations. After removing 1656 duplicates, 1596 papers were screened on title and abstract. Of these, the full texts of 91 were checked, resulting in 27 papers meeting our inclusion criteria (Table 1). No additional papers were found by contacting project members.

### General characteristics of studies

Table 1 shows the characteristics of all the studies included. A number of authors, namely Ko et al. [23, 24], McNeil et al. [25–27] and Song et al. [28, 29] discussed their own same study in several papers; each paper discussed various aspects of the study. The 27 papers that were included cover 23 different studies. All studies were published in the period 1986–2016 and published in English. Most studies were conducted in the USA (*n* = 15) or Canada (*n* = 7).

Fifteen studies had qualitative designs, generally using semi-structured individual interviews and focus groups. Eight studies had quantitative designs using a variety of methods, such as an e-mail survey and a review of medical records. Of these quantitative studies, five studies evaluated an intervention. The methodological quality was assessed as good for fifteen papers, moderate for nine and poor for three (Table 1).

### Setting and participants

Of all 23 studies, 12 derived data from homeless participants, nine studies from healthcare professionals engaged in caring for homeless people (including review or analysis of medical records) and two studies from both homeless participants and healthcare professionals (Additional file 3). Of the 12 studies that derived their data from homeless people and the two studies with both homeless participants and healthcare professionals, the homeless people were terminally ill in three studies (Table 1) [30–32].

Homeless people in the studies stayed or lived in a variety of settings. The most frequently mentioned were various types of shelters, e.g. drop-in shelters and homeless shelters. Other settings mentioned were support homes, housing facilities, hospitals and medical centres, healthcare programmes, palliative care services and hospices, hostels, social service agencies and sites or communities for homeless people (Table 1). Additional file 3 shows more information about the characteristics of the study populations. Most studies stated the age, sex and ethnicity of homeless participants. A large proportion of homeless participants were male, with percentages ranging between 60% and 100% of the study population. The average age of homeless participants varied between 43 and 65. In the studies that provided information about ethnicity, homeless people of several ethnic groups participated. The educational level of homeless participants, health status of homeless participants and characteristics of healthcare providers were reported less often.

### Concerns, care needs and future preferences for care and treatment of seriously ill homeless people

Table 2 shows the main results we extracted from the publications regarding concerns, care needs and future preferences of seriously ill homeless people about the end of life. The ‘concerns’ considered problems that homeless people had or issues they worried about. Concerns in the physical domain often were about serious illnesses and physical distress [29–31]. Psychological concerns were mostly related to fear of death and dying [24, 28, 29, 32–35]. Social concerns were mostly about being a burden to others [24, 28, 31, 35]. Spiritual concerns were hardly mentioned and were regularly described as consisting of fear of the unknown [31, 33]. Frequently mentioned concerns about care included homeless people expecting end-of-life care to be poor [23, 29, 36, 37].

**Table 2:** Concerns, care needs and future preferences for care and treatment among seriously ill homeless people

Concerns	Care needs	Preferences for future care and treatment
Physical domain • Concerns about serious illnesses and physical distress related to specific illnesses, e.g. heart disease, open heart surgery, multiple broken bones [28, 29, 31] • Fear of inappropriate and/or prolonged medical care and heroic treatments [28, 33] • Concerns about losing control over basic physical functions [24] • Concerns about being off medication [31] Psychological domain • Fear of death and dying, partly due to bad and lonely deaths of other homeless people [24, 28, 29, 32–34, 37] • Concerns about psychiatric disorders, in particular schizophrenia, mental illness, depression, affective disorder, anxiety, hearing voices, PTSD, bipolarity, uncontrolled anger [24, 31] • Fear of experiencing death by accident or violence [24, 33] Social domain • Concerns about being a burden on others [24, 28, 31, 35] • Fear of losing independence [24, 31, 33] • Concerns about dying alone [24, 31, 33] • Worries about relationships with friends and family, e.g. family not being notified, leaving a wife and children behind, lack of resources to cover burial costs, being alone, family may not show up [31, 33] • Fear of dying anonymously and no-one will be there to view their body [28, 33] • Fear that family may not know wishes, peers might help to a certain extent, but no assumptions of this help [33] • Concerns about being homeless [31] Spiritual domain • Fear of the unknown [31] • Fear that the death rituals for their culture may not take place [33] Care domain • Many patients had bad experiences from previous healthcare and social service encounters, homeless persons believe that care will be poor at the end of life [23, 29, 36, 37] • Concerns about lack of insurance and receiving sub-optimal treatment due to discrimination by HCP’s/insurance companies [31, 39] • Concerns about what will happen to the body after death, fear that their body will not be respected or taken care of [28, 33] • Homeless people who completed an advance direction worry more about the care they would receive if seriously ill or dying [36] • Fear of what will happen if no-one can speak for them [33] • Fear of being transferred to a nursing home [34]	Attitudes/behaviour of healthcare professionals • Homeless patients want to be treated with respect and dignity, e.g. treat patients like others, no judging/labelling, accept patients for who they are [28, 31, 38] • Physicians are preferred as decision-makers regarding end-of-life care treatment [23, 40] • Wish for companionship at the end of life, seeking relationship-centred, compassionate care [28, 39] • Acknowledging emotions; many homeless people have experienced tremendous losses in life. Intensifying of emotions could interfere with participants’ future decision-making process [39] • Providers who tell the truth [31] • Providers who respect privacy [31] • Providers should recognize cultural differences, this will serve as the basis for increasing sensitivity and trust [23] • Death and dying are perceived to be temporary matters, and many thought dwelling on the end of life situation was undesirable [23] • Patients prefer to use a GP who specializes in the care of the homeless [32] Involvement of family • Some of the homeless persons want family nearby, others (often a majority) do not want to burden their families [28, 38] • Requests for some form of social contact with family and friends and resolving remaining issues and disagreements before dying even if they were estranged [24, 32] • Participants who are not in contact with their family desire to be placed in a familiar environment where they could be surrounded by a social support network [24] Treatment/care options • Spirituality and religion are important components in defining life and death [23, 24, 33, 34] • Desire for advance care planning/documentation; this relates to several concerns (anonymity, estrangement, maintaining control, discussion with significant others), with trust as an important condition [28, 29, 33, 39] • Requests for detoxification [32] • Patients predominantly interact with GPS for prescriptions [32] • End-of-life care focus on pain control [28] • Asking how they would like to be remembered, including post-death wishes [31] After death • Explicit and detailed desires that homeless people’s bodies be laid to rest in a personally and culturally acceptable manner (due to the misconceptions and fears about body disposal) [28]	Treatment preferences • *Resuscitation:* - Almost all homeless persons expressed a preferences to receive cardiopulmonary resuscitation (CPR) in the event of cardiorespiratory arrest if there was a chance of returning to their current state of health^a^ [41] - Homeless people want resuscitation more than physicians and patients with COPD [40] - Homeless men are more likely to want resuscitation than homeless women [40] - Non-white homeless people are more likely to want resuscitation or life-sustaining treatment than white homeless people [40, 42] • *Life sustaining treatment:* - Nearly half of the homeless participants (8/17) indicated that they would want all measures taken, a smaller proportion (7/8) would prefer limited treatment [31] - Between 20% and 37% want life-sustaining treatment depending on condition (lowest in case of dependence, highest for unconsciousness [42] - 31% desired no life-sustaining treatment if dying [42] - In the scenario of a permanent coma or severe dementia, homeless people are more likely to want CPR or mechanical ventilation than physicians [40] Wishes for the dying process • A natural death (dying in sleep, no artificial medical interventions to prolong life, avoiding heroic measures such as prolonged life support without hope of functional recovery) [24, 33, 38] • Homeless people want to have their wishes represented when they become incompetent and/or dying [23, 39] • Dying peacefully, taking care of inner conflicts, being able to express love, apologizing to family and others [24] • Death without suffering [24] Proxy decision-makers • A significant proportion of homeless people named a proxy decision-maker^a^ [41] • Nearly all chosen surrogate decision-makers were not related; most often they were service providers, friends or (occasionally) romantic partners [28] • 29% to 34% of homeless participants showed a (written) preference for surrogate decision-making [42] • 87% of homeless participants named a family member as a surrogate decision-maker in their completed advance directives [42]

^a^When completing an advance directive

Care needs concerned topics about the care (including palliative care) that homeless people preferred or expected. Attitudes and behaviour of healthcare professionals was a theme that was often mentioned, in which treatment with respect and dignity was stated most often [28, 31, 38]. Needs concerning involvement of the family appeared to be somewhat variable. Some of the homeless want family nearby, others do not want to burden their families [28, 38] and some request some type of social contact with family and friends before dying even if they are estranged [24, 32]. Needs for treatment and care appeared to be an important theme; the most frequently mentioned were spirituality and religion [23, 24, 33, 34]. Although few spiritual concerns were mentioned in included studies, spirituality and religion appear to be important encouraging factors for homeless people when it comes needs for treatment and care. In addition, most mentioned was the possibility of expressing various concerns, such as anonymity, estrangement and maintaining control: advance care planning or documentation can help express these concerns [28, 29, 33, 39]. Only one study looked at the domain ‘after death’, showing an explicit and detailed desire that homeless people’s bodies be laid to rest in a personally and culturally acceptable manner [28]. ‘Preferences for future care and treatment; was where we grouped the preferences homeless people had in advance for the end of life; we grouped them into three domains, namely treatment preferences, the dying process and surrogate-decision making. Regarding the first domain ‘treatment preferences’, a lot of studies mentioned resuscitation and life-sustaining treatments, preferences were found to vary among subgroups of homeless people [31, 40–42]. In terms of the wishes for the dying process, a natural death was mentioned most often [24, 33, 38], e.g. no prolonged life support without hope of functional recovery. Lastly, surrogate decision-making appeared to be an important theme for homeless people at the end of life, in particular the naming of proxy to make decisions [28, 41, 42].

### The care provided: barriers and facilitators

Tables 3 and 4 show the results in terms of the barriers and facilitators for providing care to seriously ill homeless people. To give an overview of those barriers and facilitators, we identified and described three perspectives. The perspective called ‘barriers and facilitators relating to the homeless person’ revealed a lot of barriers and some facilitators. The most commonly mentioned barriers were themes related to receiving healthcare, such as end-of-life care not being a priority and living on a day-to-day basis [23, 26, 34, 39, 43], themes regarding social relationships such as the absence of support from family members and only having small networks [30, 33, 37], and themes about health-related and other behaviour, such as the limited insights homeless people have into their own health [32, 44]. Although studies reported more barriers than facilitators within this theme, a widely mentioned facilitator for homeless people was the importance of religious beliefs and spiritual experience [24, 28, 39].

**Table 3:** The care provided care: barriers relating to homeless people, interaction between homeless people and healthcare professionals, and healthcare professionals

Relating to the homeless people	Relating to the interaction between homeless people and healthcare professionals	Relating to the healthcare professionals
In relation to receiving healthcare • End-of-life care is not a priority; to obtain the basic necessities of survival and living on a day-to-day basis takes precedence over efforts to obtain health and/or end-of-life care [23, 26, 34, 38, 43] • Drug and/or alcohol dependence and non-disclosure of illicit drug use may lead to decreased opportunities for persons to remain in their usual abode or to receive and/or adhere to treatment at traditional end-of-life services due to anti-drug policies [26, 27, 34, 47] • Planning care activities and attending for hospital appointments is often difficult: patients frequently do not adhere to expected routines, arrangements for health service activities, GP and hospital appointments and often have to be reminded about their condition, homeless people are reluctant due to a long waiting time and/or they self-discharge [32, 34, 37] • Very late stage of seeking help and thus medical problems that are difficult to handle and multiple admissions before death [37, 43, 47] • A lot of homeless people are unwilling to accept the recommended treatment [44, 47] • Pain and symptom management of homeless persons who use illicit drugs (high levels of opioid tolerance) and specialists who are unable or unwilling due to fears that they would be liable for adverse reactions [26] • Lack of health insurance [43] In relation to social relationship • No support from family members or relatives and small networks and many without trusted peers [30, 33, 37] • A lot of homeless people who have psychiatric illness and are paranoia, refuse multiple offers of housing [44] • Travel and access to transport when living in a rural area [34] • Relationships between healthier and sicker patients are complex and sometimes manipulative to gain access to further alcohol [32] • Death and dying does affect other homeless patients [32] In relation to (health) behaviour • Limited insight into their condition or unable to acknowledge illnesses [32, 44] • Problems relating to alcohol and/or drug addiction, such as denial of addiction, bingeing, ignoring of risks of overdose [32] • Aggressive or changing behavior [32] • Unwillingness to pay attention to their personal hygiene [32]	• Feelings of being ignored, discriminated and disrespected by healthcare providers and a lack of trust and suspicion (e.g. shown disrespect, withholding of pain medication, inappropriately short hospital stays, not respecting wishes) that initially has to be overcome before any treatment could be started [26, 33, 35–38, 45] • End of life is an uncomfortable topic; some homeless persons do not want to know about their own diagnoses, do not want to talk about their health concerns or are incapable of talking comfortably about death and dying [23, 35, 36] • Barriers to achieving the level of communication and connections homeless people desired, e.g. too little time to chat with staff and volunteers because they were busy [34, 39] • Patients engage with internal services such as key and substance misuse workers but rarely with mental health or social workers [32] • Homeless people express many misperceptions and uncertainties about surrogate decision-making [28] • Homeless persons often describe their problems in a jumbled manner, understanding the most prioritized needs is thus not always easy [37]	Knowledge and skills • It is difficult for staff to determine when a patient is nearing the dying phase and to establish palliative care needs; staff members’ notions of palliative care vary and opportunities to prevent deaths are being missed [32, 33, 35, 37, 43] • Deaths of homeless patients are often sudden, staff were often upset [32, 33] • Hostel staff are often not able to plan for end-of-life care with patients [32] • Medical intake personnel (in hospital) do not know how to deal with a homeless person 47] • Little opportunity for funding or training shelter staff in palliative care [45] • Working with limited medical information [35] • Staff of healthcare services not being knowledgeable about the unique issues facing the homeless [36] • Often difficult to interpret reaction of patients suffering from mental illness and/or illicit drug use [37] • Trying to solve all of a patient’s problems at once is seldom successful [37] Organization • Access to palliative care, primary care and/or preventive services is minimal (due to competing priorities, attitude of healthcare professionals, anti-drug policies, not conforming to procedures, healthcare system’s nonadherence to harm reduction strategies, a lack of caregiver support and/or financial resources) and a significant proportion of homeless persons may be underusing healthcare [25, 27, 28, 32, 36, 38, 43, 47] • Lack of appropriate housing, beds, respite or hospice facilities and programmes and care sites for homeless people at the end of life and limited resources for providing end-of-life care [25, 28, 44, 45, 49] • Poor coordination and/or communication between secondary care and hostel staff or homeless programmes and end-of-life programmes [32, 35, 49] • Setting treatment goals according to routine guidelines were often regarded as unrealistic in this context [37] • In-shelter palliative care means more work for staff and a greater burden for a workforce already thinly stretched [45] • Cost of medications that was not covered by the benefits and had to be paid for in cash [30]

**Table 4:** The care provided care: facilitators relating to homeless people, interaction between homeless people and healthcare professionals, and healthcare professionals

Relating to the homeless people	Relating to the interaction between homeless people and healthcare professionals	Relating to the healthcare professionals
• Primacy of religious beliefs and spiritual experience or connection; religious beliefs are a core component of homeless people’s end-of-life beliefs and experiences; it provides comfort and solace through spirituality/religion [24, 28, 39] • Allow for patients to have “unscheduled” space to share their life stories and to acknowledge those stories [37] • Freedom is essential to homeless people [33] • Other homeless patients could become involved in the care of fellow residents who are unwilling to work with services [32] • Among homeless people who filled out an AD, there were increasing in plans to write down end-of-life wishes, plans to talk about these wishes with someone and less worrying about death [46]	Attitude towards homeless people • Building and establishing trusting and/or family-like relationships and contact by interacting with patients in everyday situations and staff taking a supportive and/or advocating role in encounters with other health providers [25, 32, 35, 37] • Upholding homeless residents’ dignity and maintaining pride by showing human kindness, respect, love, comfort and to name accomplishments and elements of character [29, 31, 35, 37] • Staff must never judge a homeless person as impossible, or in terms of failure, and always patiently give them a new chance [37] • Persistence to engage the patient and to keep them engaged, with a constant effort required for effective follow-up [47] Treatment of homeless people • A pragmatic approach by staff, facilitating flexible care solutions, such as the choice where to die and accepting that planned activities may not happen or need to be cancelled [25, 37, 45] • Compassionate healthcare providers who are present (e.g. not leaving the individual alone during or after death [25, 28, 37] • Staff can respect individual’s habits and needs (also if rather unconventional, friends and preferred surroundings (e.g. stay in the hostel) when they are at the end of life [32, 36] • Staff only contacting family members at the end of life if the patients so request [37] • Formulating simple messages towards patients about death and dying [37] Activities/therapies • Advance directive completion rate is higher when counsellor guided that compared to no counsellor guidance [41, 42, 46] • Low-threshold strategies have an increased capacity to deliver end–of-life care services [26, 27] • Harm reduction services (e.g. clean needle exchange, medically prescribed alcohol) are a critical point of entry to and source of end-of-life care and support for homeless people who use alcohol and/or illicit drugs and are unable to access services [25, 48] • Physical contact can enable feelings of safety and appreciation in patients (not all patients) [37] • Memorial services held by staff to give staff members and other patients or visitors a moment to remember and say farewell [37]	Knowledge and skills • Optimizing management of pain, symptoms and functional decline, e.g. by palliative care consultations [48, 49] • End–of-life care and addiction training [26] • To preserve integrity in being close to patients [37] • Treatment for symptoms and distress is often provided simultaneously with the use of illicit drugs and/or alcohol, this necessitates special skills for identification of signs and symptoms and treatment regimens [37] Organization In-shelter hospice care; without it, a large part of homeless patients might not have sought care or received services and died homeless with no pain and symptom management [48] • Costs of in-shelter hospice care are substantially less than the estimated costs of traditional care for the same patients [48]

Contrasting with the previous theme, a lot of studies in the theme ‘relating to the interaction between homeless people and healthcare professionals’ described facilitators and substantially fewer studies described barriers between homeless people and professionals. The attitudes of healthcare providers towards homeless persons proved to be a major theme, e.g. building and establishing relationships of trust [25, 32, 35, 37]. The treatment of homeless people was also reported to be an important theme as facilitator, e.g. a pragmatic and flexible approach from staff [25, 37, 45]. Furthermore, providing activities and therapies was also often mentioned as facilitator for the interaction between homeless people and healthcare professionals, e.g. counsellor-guided advance directive completion [41, 42, 46]. Feelings of being ignored, discriminated against and disrespected by healthcare providers and a lack of trust were often mentioned as barriers [26, 33, 35–38, 45]. For barriers and facilitators in the third theme, ‘relating to healthcare professionals’, substantially more barriers than facilitators were mentioned. The most frequently mentioned barriers were lack of knowledge and skills of professionals, e.g. the difficulty for staff in determining when a patient is nearing the dying phase and meeting the palliative care needs [32, 33, 35, 37]. Another barrier mentioned relating to healthcare professionals was the organization of care, e.g. minimal access to palliative care [26–28, 32, 36, 38, 47]. On the other hand, facilitators relating to the knowledge and skills of professionals such as optimizing management of pain, symptoms and functional decline were often mentioned [48, 49]. Facilitators regarding the overall organization of palliative care for homeless people were not found in many papers; one facilitator mentioned in a Canadian study by Podymow et al. was in-shelter hospice care, which also substantially lowers the costs [48].

### Recommendations for practice

A significant number of studies made evidence-based recommendations for practice regarding (palliative) care for seriously ill homeless people, themes are shown in Table 5 and more detailed information on the themes is shown in Additional file 4. Training, education and knowledge; delivering care, and overall organization appeared to be the comprehensive themes. A very often mentioned recommendation relating to training, education and knowledge was training for staff working with homeless people to provide palliative care as health deteriorates and death approaches [26, 27, 34, 36, 45]. Related to recommendations on delivering care, addressing themes related to a patient-centered approach concerning dignity and asking questions about death and dying in advance directive formats were most often mentioned [24, 31, 35–37, 42, 46]. Trustful and respectful relationships were also mentioned as a recommendation for delivering care; as well as attention for different domain of concerns of homeless people compared to healthcare providers, flexible programs and availability and support after death. Recommendations concerning overall organization of palliative care to homeless people concerned mostly the availability of accommodation, involved persons and coordination, policies and guidelines and partnering and exchange of knowledge between organizations. This included partnering social communities with the end-of-life care system, such as accessibility and availability of palliative care beds [34, 45].

**Table 5:** Themes regarding recommendations for practice

Training, education and knowledge	Delivering care	Overall organization
• Training regarding providing palliative care for (older) homeless people and their specific needs • Education about addressing preferences, advance directives, after death wishes and surrogate decision-makers	• Patient-centred approach • Trustful and respectful relationships • Reliability, experience, sensitivity and commitment of healthcare professionals • Attention to various areas of concern of homeless people • Flexible programmes and availability • Support after death	• Availability of accommodation • People involved and coordination • Hospital discharge policies • Policies and guidelines • Partnering and exchange of knowledge between organizations

## Discussion

This systematic review summarizes 23 relevant studies: 15 qualitative and eight quantitative studies. The concerns, needs, preferences and the barriers and facilitators described in these studies often concern the attitudes and behaviour of healthcare professionals. In particular, a respectful approach and respect for dignity proved to be important to homeless people for good quality palliative care. In addition, the limited knowledge and skills of professionals turned out to be important barriers in palliative care for homeless people. Related to that, recommendations in the studies included often concern a need for training, education and broadening of knowledge. This emphasis on change of attitudes and behaviour of healthcare professionals so that the needs of homeless people can be met was less apparent in the three other reviews that also concerned palliative care for homeless people [10, 16, 17].

Furthermore, many of the barriers we found in the studies proved to be related to the homeless people themselves. End-of-life care is often not a priority for them. Besides this, homeless people are often dependent on drugs, have limited insight into their condition and little support from family and relatives, which all make good palliative care extra challenging. Moreover, the views of homeless people about what is needed for good palliative care might differ from the views of healthcare providers. Hence, palliative care for homeless people needs a tailored approach and dialogue between healthcare providers and homeless people, as recently mentioned by Tobey et al. [7]. These outcomes are in line with the findings of the three other reviews [10, 16, 17].

As this review included relatively many studies and the methodological quality of the majority of studies was rated as good, it provides good insights into what is presently known with regard to palliative care for homeless people. At the same time, the review also sheds light on gaps in that knowledge. A large majority of the studies were conducted in the USA and Canada. More studies from other countries are needed as it is very well possible that differences in culture, the organization of homeless care and the organization of healthcare could lead to different results for different countries. It remains for instance to be seen whether spirituality and religion – which proved very important to homeless people in this study – will be as important in more secular countries such as the Netherlands. Furthermore, the studies that had qualitative designs often provide important insights into the experiences and ideas of homeless people and their care providers that are helpful in initiatives aimed at improving the care. Although this review mentioned that homeless people get minimal access to palliative care, primary care and/or preventive services, no details are known about homeless people who completely avoid care. If healthcare providers want to provide tailored palliative care to the entire target group, more research is needed into palliative care for homeless people who avoid care. Because the homeless people who avoid care are hard to reach, it is advisable to do participatory observation or to interview people who use successful methods to reach them, such as street pastors. Finally, more information is needed about healthcare providers who provide palliative care to homeless people. The studies included mostly concerned characteristics of homeless people, but little is known about the background characteristics in terms of the experience and preferences of healthcare providers. Future studies can study the healthcare professionals. This can help provide tailored training, education and knowledge for healthcare providers.

Our review also included intervention studies that provide information about interventions for tailoring palliative care to the needs of homeless people. Several studies indicated advance care planning and documentation as a potentially effective way of encountering the concerns and needs of homeless people, such as a fear of death, anonymity, estrangement, maintaining control and discussions with significant others. These studies were also included in the review by Sumalinog et al. that focused on interventions [10]. In that review, the methodology of these studies was rated at between poor and fair, which is lower than our methodological ratings. This can be explained by the fact that we used an assessment tool that can be used for various types of studies, while Sumalinog et al. used a tool that was appropriate for assessing whether intervention studies provide strong evidence for the intervention being effective. While this shows that there is no strong evidence for the interventions being effective, the results of these studies can provide pointers to help develop new interventions and study them thoroughly.

### Strengths and limitations

One strength of this systematic review is that it looks at the concerns, care needs and preferences, barriers and facilitators and recommendations for practice, thereby providing a broad overview of topics that are relevant to palliative care for homeless people. In addition, the broad inclusion criteria resulted in a large number of studies being included (given the limited size of the field being researched). Moreover, this review combines both qualitative and quantitative studies. Finally, another strength of this systematic review is that doing a grey literature search meant that we also included studies by organizations working in the field, such as Simon Communities and St. Mungo’s.

An initial limitation of this study is that the definition and terminology of palliative and/or end-of-life care differ according to the study. Studies may therefore include other aspects of palliative or end-of-life care while using the same definition and terminology. A second limitation is that both studies of seriously ill homeless people and studies of homeless people who expressed their expectations about being seriously ill have been included. Expectations about the end of life in advance may differ from the reality later. Another limitation was that, although we aimed only to summarize the recommendations from the studies’ results, it is was not always certain that the recommendations were not also reflections of the author’s opinions. As a fourth limitation, this systematic review lacks studies in published in languages other than English. Finally, a methodological limitation was that in some studies it was difficult to assess the methodological quality because some information was missing. It is possible that in those cases the actual study was conducted in a more thorough way than reported on in the article.

### Conclusions

Homeless people at the end of life experience a range of problems and barriers concerning access to palliative care. A tailored, flexible and low-threshold approach consisting of awareness about the fear of death among homeless people (as well as priorities and needs of homeless people other than those assessed by healthcare professionals) can be used to help provide appropriate care in good time. This tailored, flexible and low-threshold approach should at least involve awareness of the concerns of homeless people (fear of death and negative experiences with healthcare providers). This requires sensitivity and patience of healthcare professionals. In addition, awareness about the meaning of dignity and respect to the homeless patient is important when it comes to understanding the needs of homeless people, as well as recognizing important components such as religiosity and documentation of future preferences. Finally, healthcare professionals need to be aware that future preferences may be different for homeless patients compared to a non-homeless patient, and therefore ask specific questions about it. Training, education and experience of healthcare providers can accomplish this.

## Additional files

Additional file 1: Search strategies. (DOCX 28 kb)

Additional file 2: Details of assessments of studies by using the Critical Appraisal Tool. (DOCX 22 kb)

Additional file 3: Characteristics of study populations. (DOCX 29 kb)

Additional file 4: Recommendations for practice. (DOCX 30 kb)

## Abbreviations

AIDS: Acquired immune deficiency syndrome
COPD: Chronic obstructive pulmonary disease
PRISMA: Preferred reporting items for systematic reviews and meta-analysis
WHO: World Health Organization
ZonMw: The Netherlands Organisation for Health Research and Development
