# Supplementary material for: Palliative care for homeless people: a systematic review of the concerns, care needs and preferences, and the barriers and facilitators for providing palliative care
Source: BMC Palliat Care. 2018 Apr 24;17:67. doi: 10.1186/s12904-018-0320-6 (PMC5914070; doi:10.1186/s12904-018-0320-6)
Supplement: Supplementary file 1 — Search strategies. (DOCX 28 kb) [file 12904_2018_320_MOESM1_ESM.docx]

# Appendix 1: Search strategies

/exp = EMtree keyword with explosion

:ab,ti = words in title or abstract

NEAR/3 = words near to each other, 3 places apart

Search strategy for Embase.com (April 1, 2016)

| Search | Query | Items found |
| --- | --- | --- |
| #3 | #1 AND #2 | 899 |
| #2 | 'terminal care'/de OR 'hospice care'/exp OR 'palliative therapy'/de OR 'palliative nursing'/exp OR 'hospice'/exp OR 'hospice nursing'/exp OR 'death'/de OR 'dying'/exp OR 'attitude to death'/exp OR 'mortality'/de OR terminal*:ab,ti OR 'end of life':ab,ti OR 'life care end':ab,ti OR hospice*:ab,ti OR (bereavement NEAR/3 car*):ab,ti OR palliati*:ab,ti OR (limited NEAR/3 life*):ab,ti OR death*:ab,ti OR dying*:ab,ti OR die:ab,ti OR mortal*:ab,ti OR 'advanced car*':ab,ti OR 'advance car*':ab,ti | 2,358,671 |
| #1 | 'homelessness'/exp OR homeless*:ab,ti OR 'street people*':ab,ti | 11,028 |

Search strategy for Ebsco/PsycInfo (April 1, 2016)
DE = descriptors, keywordsTI = words in title
AB = words in abstract
N3 = words near to each other, 3 places apart

| Search | Query | Items found |
| --- | --- | --- |
| S3 | S1 AND S2 | 332 |
| S2 | ( DE "Terminally Ill Patients" OR DE "Advance Directives" OR DE "Death and Dying" OR DE "Hospice" OR DE "Palliative Care" OR DE "Death Attitudes" ) OR TI ( terminal* OR “end of life” OR “life care end” OR hospice* OR (bereavement N3 car*) OR palliati* OR (limited N3 life*) OR death* OR dying* OR die OR mortal* OR “advanced car*” OR “advance car*” ) OR AB ( terminal* OR “end of life” OR “life care end” OR hospice* OR (bereavement N3 car*) OR palliati* OR (limited N3 life*) OR death* OR dying* OR die OR mortal* OR “advanced car*” OR “advance car*” ) | 145,273 |
| S1 | DE "Homeless" OR TI ( homeless* OR “street people*” ) OR AB ( homeless* OR “street people*” ) | 8,750 |

Search strategy for Ebsco/CINAHL (April 5, 2016)
MH = keywords
TI = words in title
AB = words in abstract
N3 = words near to each other, 3 places apart

| Search | Query | Items found |
| --- | --- | --- |
| S3 | S1 AND S2 | 283 |
| S2 | ( (MH "Terminal Care (Saba CCC)") OR (MH "Terminal Care") OR (MH "Palliative Care") OR (MH "Hospice and Palliative Nursing") OR (MH "Hospice Care") OR (MH "Death") OR (MH "Dying Care (Iowa NIC)") OR (MH "Attitude to Death") OR (MH "Advance Care Planning") ) OR TI ( terminal* OR “end of life” OR “life care end” OR hospice* OR (bereavement N3 car*) OR palliati* OR (limited N3 life*) OR death* OR dying* OR die OR mortal* OR “advanced car*” OR “advance car*” ) OR AB ( terminal* OR “end of life” OR “life care end” OR hospice* OR (bereavement N3 car*) OR palliati* OR (limited N3 life*) OR death* OR dying* OR die OR mortal* OR “advanced car*” OR “advance car*” ) | 170,379 |
| S1 | ( (MH "Homeless Persons") OR (MH "Homelessness") ) OR TI ( homeless* OR “street people*” ) OR AB ( homeless* OR “street people*” ) | 6,233 |

Search strategy for Thomson Reuters/Web of Science (May 3, 2016)
TOPIC = words in title, abstract or keywords
NEAR/3 = words near to each other, 3 places apart
Indexes=SCI-EXPANDED, SSCI, A&HCI, ESCI; Timespan=All years

| Search | Query | Items found |
| --- | --- | --- |
| #3 | #2 AND #1 | 1,116 |
| #2 | TOPIC: (terminal* OR “end of life” OR “life care end” OR hospice* OR (bereavement NEAR/3 car*) OR palliati* OR (limited NEAR/3 life*) OR death* OR dying* OR die OR mortal* OR “advanced car*” OR “advance car*”) | 4,071,753 |
| #1 | TOPIC: (homeless* OR “street people*”) | 21,929 |

Search strategy for PubMed (May 10, 2016)
[Mesh] = Medical subject headings (MeSH)
[Mesh:NoExp] = Medical subject headings (MeSH) without explosion
[Subheading] = qualifier to MeSH term
[tiab] = words in title or abstract

| Search | Query | Items found |
| --- | --- | --- |
| #3 | #1 AND #2 | 614 |
| #2 | "Terminal Care"[Mesh] OR "Palliative Care"[Mesh] OR "Palliative Medicine"[Mesh] OR "Hospice and Palliative Care Nursing"[Mesh] OR "Death"[Mesh:NoExp] OR "mortality"[Subheading] OR terminal*[tiab] OR end of life[tiab] OR life care end[tiab] OR hospice*[tiab] OR bereavement car*[tiab] OR palliati*[tiab] OR limited life*[tiab] OR death*[tiab] OR dying*[tiab] OR die[tiab] OR "Advance Care Planning"[Mesh] OR “Attitude to death”[Mesh] OR mortal*[tiab] OR advanced car*[tiab] OR advance car*[tiab] | 1,797,180 |
| #1 | "Homeless Persons"[Mesh:NoExp] OR homeless*[tiab] OR street people*[tiab] | 9,277 |
