# Supplementary material for: Palliative care for homeless people: a systematic review of the concerns, care needs and preferences, and the barriers and facilitators for providing palliative care
Source: BMC Palliat Care. 2018 Apr 24;17:67. doi: 10.1186/s12904-018-0320-6 (PMC5914070; doi:10.1186/s12904-018-0320-6)
Supplement: Supplementary file 2 — Details of assessments of studies by using the Critical Appraisal Tool. (DOCX 22 kb) [file 12904_2018_320_MOESM2_ESM.docx]

## Appendix 4. Details of assessments of studies by using the Critical Appraisal Tool.

| **Study** | **Reviewer 1 (HTK)** | **Reviewer 2 (AJEV)** |
| --- | --- | --- |
| Bartels et al. [31] | Abstract and title: very poor (1)  Introduction and aims: fair (3)  Method and data: good (4)  Sampling: fair (3)  Data-analysis: poor (2)  Ethics and bias: poor (2)  Findings/results: fair (3)  Transferability/generalizability: fair (3)  Implications and usefulness: fair (3)  Total = 24 (moderate) | Abstract and title: very poor (1)  Introduction and aims: fair (3)  Method and data: fair (3)  Sampling: fair (3)  Data-analysis: poor (2)  Ethics and bias: very poor (1)  Findings/results: fair (3)  Transferability/generalizability: poor (2)  Implications and usefulness: poor (2)  Total = 20 (moderate) |
| Davis et al. [32] | Abstract and title: very poor (1)  Introduction and aims: fair (3)  Method and data: good (4)  Sampling: fair (3)  Data-analysis: poor (2)  Ethics and bias: very poor (1)  Findings/results: fair (3)  Transferability/generalizability: poor (2)  Implications and usefulness: fair (3)  Total = 22 (moderate) | Abstract and title: poor (2)  Introduction and aims: poor (2)  Method and data: fair (3)  Sampling: good (4)  Data-analysis: poor (2)  Ethics and bias: very poor (1)  Findings/results: poor (2)  Transferability/generalizability: poor (2)  Implications and usefulness: fair (3)  Total = 21 (moderate) |
| Hakanson et al. [37] | Abstract and title: good (4)  Introduction and aims: good (4)  Method and data: good (4)  Sampling: good (4)  Data-analysis: fair (3)  Ethics and bias: fair (3)  Findings/results: good (4)  Transferability/generalizability: good (4)  Implications and usefulness: good (4)  Total = 34 (good) | Abstract and title: fair (3)  Introduction and aims: good (4)  Method and data: fair (3)  Sampling: good (4)  Data-analysis: fair (3)  Ethics and bias: fair (3)  Findings/results: good (4)  Transferability/generalizability: fair (3)  Implications and usefulness: good (4)  Total = 31 |
| Hutt et al. [49] | Abstract and title: good (4)  Introduction and aims: fair (3)  Method and data: fair (3)  Sampling: fair (3)  Data-analysis: good (4)  Ethics and bias: poor (2)  Findings/results: good (4)  Transferability/generalizability: poor (2)  Implications and usefulness: fair (3)  Total = 28 (moderate) | Abstract and title: fair (3)  Introduction and aims: fair (3)  Method and data: poor (2)  Sampling: poor (2)  Data-analysis: fair (3)  Ethics and bias: fair (3)  Findings/results: poor (2)  Transferability/generalizability: poor (2)  Implications and usefulness: fair (3)  Total = 23 (moderate) |
| Hwang et al. [43] | Abstract and title: poor (2)  Introduction and aims: fair (3)  Method and data: fair (3)  Sampling: good (4)  Data-analysis: fair (3)  Ethics and bias: very poor (1)  Findings/results: good (4)  Transferability/generalizability: fair (3) Implications and usefulness: good (3)  Total = 27 (moderate) | Abstract and title: poor (2)  Introduction and aims: poor (2)  Method and data: poor (2)  Sampling: good (4)  Data-analysis: good (4)  Ethics and bias: very poor (1)  Findings/results: good (4)  Transferability/generalizability: fair (3)  Implications and usefulness: fair (3)  Total = 25 (moderate) |
| Ko et al. [23] | Abstract and title: fair (3)  Introduction and aims: fair (3)  Method and data: fair (3)  Sampling: fair (3)  Data-analysis: good (4)  Ethics and bias: good (4)  Findings/results: good (4)  Transferability/generalizability: fair (3)  Implications and usefulness: good (4)  Total = 30 (good) | Abstract and title: poor (2)  Introduction and aims: fair (3)  Method and data: good (4)  Sampling: good (4)  Data-analysis: good (4)  Ethics and bias: poor (2)  Findings/results: good (4)  Transferability/generalizability: poor (2)  Implications and usefulness: good (4)  Total = 29 (good) |
| Ko et al. [24] | Abstract and title: poor (2)  Introduction and aims: good (4)  Method and data: fair (3)  Sampling: fair (3)  Data-analysis: good (4)  Ethics and bias: good (4)  Findings/results: good (4)  Transferability/generalizability: poor (2)  Implications and usefulness: good (4)  Total = 30 (good) | Abstract and title: fair (3)  Introduction and aims: good (4)  Method and data: good (4)  Sampling: good (4)  Data-analysis: good (4)  Ethics and bias: good (4)  Findings/results: good (4)  Transferability/generalizability: poor (2)  Implications and usefulness: good (4)  Total = 33 (good) |
| Krakowsky et al. [36] | Abstract and title: fair (3)  Introduction and aims: poor (2)  Method and data: poor (2)  Sampling: poor (2)  Data-analysis: very poor (1)  Ethics and bias: poor (2)  Findings/results: poor (2)  Transferability/generalizability: poor (2)  Implications and usefulness: fair (3)  Total = 19 (poor) | Abstract and title: fair (3)  Introduction and aims: poor (2)  Method and data: poor (2)  Sampling: poor (2)  Data-analysis: very poor (1)  Ethics and bias: poor (2)  Findings/results: poor (2)  Transferability/generalizability: very poor (1)  Implications and usefulness: fair (3)  Total = 18 (poor) |
| Leung et al. [41] | Abstract and title: good (4)  Introduction and aims: good (4)  Method and data: fair (3)  Sampling: fair (3)  Data-analysis: good (4)  Ethics and bias: fair (3)  Findings/results: good (4)  Transferability/generalizability: poor (2)  Implications and usefulness: good (4)  Total = 31 (good) | Abstract and title: good (4)  Introduction and aims: good (4)  Method and data: fair (3)  Sampling: good (4)  Data-analysis: good (4)  Ethics and bias: fair (3)  Findings/results: good (4)  Transferability/generalizability: fair (3)  Implications and usefulness: good (4)  Total = 33 (good) |
| Mac Williams et al. [47] | Abstract and title: fair (3)  Introduction and aims: good (4)  Method and data: fair (3)  Sampling: fair (4)  Data-analysis: poor (2)  Ethics and bias: fair (3)  Findings/results: fair (3)  Transferability/generalizability: poor (2)  Implications and usefulness: good (4)  Total = 27 (moderate) | Abstract and title: fair (3)  Introduction and aims: good (4)  Method and data: poor (2)  Sampling: poor (2)  Data-analysis: poor (2)  Ethics and bias: poor (2)  Findings/results: fair (3)  Transferability/generalizability: poor (2)  Implications and usefulness: good (4)  Total = 23 (moderate) |
| McGrath [30] | Abstract and title: poor (2)  Introduction and aims: good (4)  Method and data: good (4)  Sampling: poor (2)  Data-analysis: very poor (1)  Ethics and bias: very poor (1)  Findings/results: fair (3)  Transferability/generalizability: very poor (1)  Implications and usefulness: fair (3)  Total = 21 (moderate) | Abstract and title: fair (3)  Introduction and aims: good (4)  Method and data: poor (2)  Sampling: poor (2)  Data-analysis: very poor (1)  Ethics and bias: very poor (1)  Findings/results: very poor (1)  Transferability/generalizability: poor (2)  Implications and usefulness: fair (3)  Total = 19 (moderate) |
| McNeil & Guirguis-Yonger [25] | Abstract and title: fair (3)  Introduction and aims: good (4)  Method and data: good (4)  Sampling: poor (2)  Data-analysis: good (4)  Ethics and bias: poor (2)  Findings/results: good (4)  Transferability/generalizability: poor (2)  Implications and usefulness: good (4)  Total = 29 (good) | Abstract and title: poor (2)  Introduction and aims: good (4)  Method and data: fair (3)  Sampling: fair (3)  Data-analysis: good (4)  Ethics and bias: good (4)  Findings/results: good (4)  Transferability/generalizability: fair (3)  Implications and usefulness: good (4)  Total = 31 (good) |
| McNeil et al. [26] | Abstract and title: good (4)  Introduction and aims: fair (3)  Method and data: good (4)  Sampling: fair (3)  Data-analysis: good (4)  Ethics and bias: fair (3)  Findings/results: good (4)  Transferability/generalizability: fair (3)  Implications and usefulness: good (4)  Total = 32 (good) | Abstract and title: good (4)  Introduction and aims: good (4)  Method and data: good (4)  Sampling: good (4)  Data-analysis: good (4)  Ethics and bias: good (4)  Findings/results: good (4)  Transferability/generalizability: fair (3)  Implications and usefulness: good (4)  Total = 35 (good) |
| McNeil et al. [24] | Abstract and title: good (4 Introduction and aims: fair (3)  Method and data: good (4)  Sampling: fair (3)  Data-analysis: good (4)  Ethics and bias: fair (3)  Findings/results: good (4)  Transferability/generalizability: fair (3)  Implications and usefulness: good (4)  Total = 32 (good) | Abstract and title: good (4)  Introduction and aims: good (4)  Method and data: fair (3)  Sampling: fair (3)  Data-analysis: good (4)  Ethics and bias: good (4)  Findings/results: good (4)  Transferability/generalizability: fair (3)  Implications and usefulness: good (4)  Total = 33 (good) |
| Nikouline & Dosani [45] | Abstract and title: very poor (1)  Introduction and aims: fair (3)  Method and data: fair (3)  Sampling: poor (2)  Data-analysis: fair (3)  Ethics and bias: very poor (1)  Findings/results: good (4)  Transferability/generalizability: poor (2)  Implications and usefulness: good (4)  Total = 23 (moderate) | Abstract and title: very poor (1)  Introduction and aims: poor (2)  Method and data: poor (2)  Sampling: poor (2)  Data-analysis: poor (2)  Ethics and bias: poor (2)  Findings/results: fair (3)  Transferability/generalizability: poor (2)  Implications and usefulness: fair (3)  Total = 19 (moderate) |
| Norris et al. [40] | Abstract and title: good (4)  Introduction and aims: good (4)  Method and data: fair (3)  Sampling: good (4)  Data-analysis: good (4)  Ethics and bias: poor (2)  Findings/results: good (4)  Transferability/generalizability: fair (3)  Implications and usefulness: fair (3)  Total = 31 (good) | Abstract and title: good (4)  Introduction and aims: good (4)  Method and data: fair (3)  Sampling: fair (3)  Data-analysis: good (4)  Ethics and bias: fair (3)  Findings/results: good (4)  Transferability/generalizability: fair (3)  Implications and usefulness: good (4)  Total = 32 (good) |
| O’Connell et al. [44] | Abstract and title: fair (3)  Introduction and aims: very poor (1)  Method and data: very poor (1)  Sampling: poor (2)  Data-analysis: very poor (1)  Ethics and bias: very poor (1)  Findings/results: fair (3)  Transferability/generalizability: poor (2)  Implications and usefulness: very poor (1)  Total = 15 (poor) | Abstract and title: poor (2)  Introduction and aims: very poor (1)  Method and data: very poor (1)  Sampling: poor (2)  Data-analysis: very poor (1)  Ethics and bias: very poor (1)  Findings/results: fair (3)  Transferability/generalizability: very poor (1)  Implications and usefulness: very poor (1)  Total = 13 |
| Podymow et al. [47] | Abstract and title: good (4)  Introduction and aims: good (4) Method and data: good (4) Sampling: good (4) Data-analysis: good (4) Ethics and bias: poor (2)  Findings/results: good (4) Transferability/generalizability: good (4)  Implications and usefulness: poor (2)  Total = 32 (good) | Abstract and title: good (4)  Introduction and aims: poor (2)  Method and data: fair (3)  Sampling: good (4)  Data-analysis: good (4)  Ethics and bias: poor (2)  Findings/results: good (4)  Transferability/generalizability: good (4)  Implications and usefulness: fair (3)  Total = 30 (good) |
| Ratner et al. [38] | Abstract and title: poor (2)  Introduction and aims: poor (2)  Method and data: poor (2)  Sampling: fair (3)  Data-analysis: poor (2)  Ethics and bias: very poor (1)  Findings/results: poor (2)  Transferability/generalizability: poor (2)  Implications and usefulness: poor (2)  Total = 18 (poor) | Abstract and title: poor (2)  Introduction and aims: fair (3)  Method and data: poor (2)  Sampling: fair (3)  Data-analysis: very poor (1)  Ethics and bias: very poor (1)  Findings/results: poor (2)  Transferability/generalizability: poor (2)  Implications and usefulness: poor (2)  Total = 18 (poor) |
| Song et al. [33] | Abstract and title: very poor (1)  Introduction and aims: fair (3)  Method and data: fair (3)  Sampling: poor (2)  Data-analysis: fair (3)  Ethics and bias: poor (2)  Findings/results: good (4)  Transferability/generalizability: poor (2)  Implications and usefulness: fair (3)  Total = 23 (moderate) | Abstract and title: very poor (1)  Introduction and aims: poor (2)  Method and data: fair (3)  Sampling: poor (2)  Data-analysis: fair (3)  Ethics and bias: fair (3)  Findings/results: good (4)  Transferability/generalizability: poor (2)  Implications and usefulness: good (4)  Total = 24 (moderate) |
| Song et al. [29] | Abstract and title: good (4)  Introduction and aims: good (4)  Method and data: fair (3)  Sampling: fair (3)  Data-analysis: fair (3)  Ethics and bias: fair (3)  Findings/results: fair (3)  Transferability/generalizability: fair (3)  Implications and usefulness: good (4)  Total = 30 (good) | Abstract and title: good (4)  Introduction and aims: good (4)  Method and data: good (4)  Sampling: good (4)  Data-analysis: good (4)  Ethics and bias: good (4)  Findings/results: good (4)  Transferability/generalizability: fair (3)  Implications and usefulness: good (4)  Total = 35 (good) |
| Song et al. [28] | Abstract and title: good (4)  Introduction and aims: good (4)  Method and data: fair (3)  Sampling: fair (3) Data-analysis: fair (3) Ethics and bias: fair (3) Findings/results: fair (3) Transferability/generalizability: fair (3) Implications and usefulness: fair (3) Total = 29 (good) | Abstract and title: good (4)  Introduction and aims: good (4)  Method and data: fair (3)  Sampling: good (4)  Data-analysis: good (4)  Ethics and bias: fair (3)  Findings/results: fair (3)  Transferability/generalizability: fair (3)  Implications and usefulness: good (4)  Total = 32 (good) |
| Song et al. [46] | Abstract and title: good (4)  Introduction and aims: good (4)  Method and data: good (4)  Sampling: fair (3) Data-analysis: fair (3) Ethics and bias: good (4)  Findings/results: fair (3) Transferability/generalizability: fair (3) Implications and usefulness: good (4)  Total = 32 (good) | Abstract and title: good (4)  Introduction and aims: good (4)  Method and data: good (4)  Sampling: good (4)  Data-analysis: good (4)  Ethics and bias: good (4)  Findings/results: good (4)  Transferability/generalizability: fair (3)  Implications and usefulness: good (4)  Total = 35 (good) |
| Song et al. [42] | Abstract and title: good (4)  Introduction and aims: fair (3)  Method and data: good (4)  Sampling: good (4)  Data-analysis: fair (3) Ethics and bias: poor (2)  Findings/results: good (4)  Transferability/generalizability: fair (3) Implications and usefulness: good (4)  Total = 31 (good) | Abstract and title: good (4)  Introduction and aims: good (4)  Method and data: good (4)  Sampling: fair (3)  Data-analysis: good (4)  Ethics and bias: good (4)  Findings/results: good (4)  Transferability/generalizability: good (4)  Implications and usefulness: good (4)  Total = 35 (good) |
| Tarzian et al. [39] | Abstract and title: good (4)  Introduction and aims: fair (3)  Method and data: good (4)  Sampling: good (4)  Data-analysis: poor (2)  Ethics and bias: fair (3)  Findings/results: good (4)  Transferability/generalizability: fair (3) Implications and usefulness: good (4)  Total = 31 (good) | Abstract and title: good (4)  Introduction and aims: fair (3)  Method and data: good (4)  Sampling: fair (3)  Data-analysis: fair (3)  Ethics and bias: fair (3)  Findings/results: good (4)  Transferability/generalizability: good (4)  Implications and usefulness: good (4)  Total = 28 (good) |
| Walsh [34] | Abstract and title: very poor (1)  Introduction and aims: good (4)  Method and data: poor (2)  Sampling: poor (2)  Data-analysis: very poor (1)  Ethics and bias: good (4)  Findings/results: fair (3)  Transferability/generalizability: very poor (1)  Implications and usefulness: good (4)  Total = 22 (moderate) | Abstract and title: very poor (1)  Introduction and aims: good (4)  Method and data: fair (3)  Sampling: poor (2)  Data-analysis: very poor (1)  Ethics and bias: fair (3)  Findings/results: fair (3)  Transferability/generalizability: poor (2)  Implications and usefulness: fair (3)  Total = 22 (moderate) |
| Webb [35] | Abstract and title: good (4)  Introduction and aims: fair (3)  Method and data: good (4)  Sampling: poor (2)  Data-analysis: good (4)  Ethics and bias: fair (3)  Findings/results: fair (3)  Transferability/generalizability: poor (2)  Implications and usefulness: fair (3)  Total = 28 (good) | Abstract and title: good (4)  Introduction and aims: good (4)  Method and data: good (4)  Sampling: fair (3)  Data-analysis: good (4)  Ethics and bias: fair (3)  Findings/results: good (4)  Transferability/generalizability: fair (3)  Implications and usefulness: poor (2)  Total = 33 (good) |
