# Supplementary material for: Palliative care for homeless people: a systematic review of the concerns, care needs and preferences, and the barriers and facilitators for providing palliative care
Source: BMC Palliat Care. 2018 Apr 24;17:67. doi: 10.1186/s12904-018-0320-6 (PMC5914070; doi:10.1186/s12904-018-0320-6)
Supplement: Supplementary file 3 — Characteristics of study populations. (DOCX 29 kb) [file 12904_2018_320_MOESM3_ESM.docx]

# Appendix 2. Characteristics of study populations (categorization as used in the original studies).

| Reference | Type of participants | Sex (% male) | Age (m) | Cultural background | Health status | Education |
| --- | --- | --- | --- | --- | --- | --- |
| [28] | Homeless people | 82% | NA | 64.7% Black  23.5% Non-Hispanic White  11.8% Native American | NA | 41.2% Finished college 35.3% High school graduates  23.5% No answer |
| [23, 24] | Homeless people | 86% | 65 | 52.4% White  23.8% Black  14.3% Latino/Hispanics  9.5% Other | 57.1% (Very) good  23.8% Fair  19% Poor | 57.1% College education  28.6% High school graduates 9.5% Less than high school 4.8% Post graduate |
| [41] | Homeless people | 100% | 55 | 70.2% White  8.8% Black  6.8% Aboriginal  11.2% Asian 2.9% Other | 7.8% Excellent 15.7% Very good 32.8% Good 25.5% Fair 18.1% Poor | 12.2% 8^th^ grade or lower 38% High school 21.5% High school diploma 16.1% College or university 12.2% Bachelor’s degree |
| [47] | Homeless people | NA | NA | NA | NA | NA |
| [32] | Homeless people | 100% | 62 | 75% Caucasian  13% Black  13% Hispanic |  | 25% Started college  75% Unknown[ |
| [38] | Homeless people | NA | 48 | 40% American Indians  29% Black | NA | 34% More than 12 years education |
| [28, 29] | Homeless people | 66% | 47 | 36% American Indian or Alaskan Native 27% Black  22% White 7% Not reported 2% Hispanic or Latino 2% Not Hispanic or Latino 2% Asian  2% Native African | NA | Years of education:  8% 5-8 39% 9-11 32% 12-15 8% >16 13% Not reported |
| [46] | Homeless people | 75% | 45 | 34% White  51% Black  7% Native American  3% Hispanic  2% Asian  3% Mixed | NA | 11% <High School  48% High School  40% >High School |
| [42] | Homeless people | 74% | 43 | 31.7% White  53.8% Black  6,9% Native American  0.8% Asian  97.3%Non-Hispanic  2.7% Hispanic | NA | 30.6% Did not complete high school  69.4% High school or higher |
| [39] | Homeless people | 60% | NA | 80% African American  20% Caucasian | NA | NA |
| [34] | Homeless people | 81% | 61 | NA | NA | NA |
| [48] | Homeless people | 89% | 49 | 89.3% Caucasian  7.9% African American  3.6% Aboriginal | NA | NA |
| [40] | Homeless people and healthcare professionals* | 59% | 51 | 40.7% White  37% African American  10.4% Hispanic  3.7% Asian 8.1% Native American | 8.1% Excellent  15.6% Very good  31.1% Good  25.2% Fair | 1.5% No formal education  3.7% <8^th^ grade 14.7% High school  25.2% High school graduate  44.4% College/trade school 8.9% College degree  1.5% Graduate degree |
| [33] | Homeless people and healthcare professionals | NA | NA | NA | NA | NA |
| [25, 26, 27] | Healthcare professionals | NA | NA | NA | NA | NA |
| [43] | Healthcare professionals*, ** | 86% | 47 | 59.1% White  30.3% Black  8.1% Hispanic  2.5% Other |  |  |
| [44] | Healthcare professionals*, ** | 73% | 67 | NA | NA | NA |
| [32] | Healthcare professionals*, ** | 96% | 55 | 70.4% White British  14.8% Irish 7.4% Black 3.7% Portuguese  3.7% Afro Caribbean | NA | NA |
| [45] | Healthcare professionals | NA | NA | NA | NA | NA |
| [37] | Healthcare professionals | NA | NA | NA | NA | NA |
| [49] | Healthcare professionals | NA | NA | NA | NA | NA |
| [36] | Healthcare professionals | NA | NA | NA | NA | NA |
| [35] | Healthcare professionals | NA | NA | NA | NA | NA |

NA = not applicable
*Only data available of homeless persons
** Data was collected by healthcare providers but provides information about homeless persons, e.g. a cohort or review of medical files
