# Supplementary material for: Palliative care for homeless people: a systematic review of the concerns, care needs and preferences, and the barriers and facilitators for providing palliative care
Source: BMC Palliat Care. 2018 Apr 24;17:67. doi: 10.1186/s12904-018-0320-6 (PMC5914070; doi:10.1186/s12904-018-0320-6)
Supplement: Supplementary file 4 — Recommendations for practice. (DOCX 30 kb) [file 12904_2018_320_MOESM4_ESM.docx]

# Appendix 3. Recommendations for practice

| Training, education and knowledge | Delivering care | Overall organization |
| --- | --- | --- |
| *Training and education regarding providing palliative care to and specific needs of (older) homeless people*  • Training for staff working with homeless people to provide support as health deteriorates and death approaches, e.g. talking about death and dying, talking about the unique needs of the homeless population, pain and symptom management, substance use and recognition of health needs [26, 27, 34-36, 45]  *Training and education about addressing preferences, ACP, advance directives, after-death wishes and surrogate decision-makers* • The importance of religion/spirituality in the participant’s attitudes towards ACP needs to be recognized, a holistic approach that incorporates a religious/spiritual component as a part of ACP process will be necessary [23, 34] • Having some form of identification card with simple directives or contacts may also serve to preserve the dignity and autonomy of homeless persons at the end of life [33] • Homeless individuals’ levels of motivation and perceived ability to engage in ACP might vary with their circumstances, so preparing them to consider death and dying might be an important first step to take. In doing so, engaging in EOL communication gradually and over time can help reach better future decisions [23] *Training and educational initiatives directed towards both providers and homeless clients* • Educational initiatives directed towards both providers and homeless clients may be extremely beneficial, end-of-life service providers need to understand the end–of-life concerns of their clients, and toward hospice and other end-of-life care and healthcare providers who need to understand the special concerns and needs of homeless individuals [26, 27, 33]  *Access to support that acknowledges the challenges of professionals*  • Access to bereavement support that acknowledges the challenges faced by staff and other residents in coming to terms with unanticipated deaths [32] | *Patient-centred approach* • A patient-centered approach: respecting each persons’ individual story, autonomy, dignity and own end-of-life priorities, wishes and desires [24, 31, 35-37, 42, 46]  • Harm reduction approaches and interventions that have minimal requirements for admission and care are needed to improve health equity and promote dignity for homeless persons who use illicit drugs at the end of life [25-27, 36, 48] • A non-judgmental, culturally sensitive and individually tailored approach, deviating from policies and guidelines should be more the rule rather than the exception [23, 26, 37] • Decision-making for homeless persons by physicians should not be made based on assumptions or physicians’ preferences [38, 40]  *Trusting and respectful relationships* • Contextual, experience-based skills that go beyond palliative care education and should be based on trusting and respectful relationships between provider and patient and on previous encounters [36, 37, 43]  • Healthcare professionals who help homeless people to rebuild and strengthen social support [24] • A “professional family member” for those estranged from their family can support patients who are homeless in their emotional, physical and social struggles [37] • Healthcare professionals or patient navigators might serve as an advocate for homeless persons as they try to navigate the end-of- life care system and help minimize the impact of discrimination and/or exclusionary policies [27, 36] • Expand individuals’ social networks (using volunteers/former service users) and community mobilization [34]  *Reliability, experience and sensitivity of healthcare professionals* • Health practitioners needed with strong communication skills, this shows reliability and commitment to the homeless population [45] • Healthcare providers need to be sensitive and respond effectively to varying emotional responses that may stem from fear of pain, suffering, and losing control in EOL situations [24]  *Attention for various areas of concern of homeless people* • Areas of concern of homeless people at the end of life are different to those of the general population and require a different approach; healthcare providers should be aware of a deep level of concern about morbidity, premature mortality, and decisions they might confront as they approach the end of their lives. Listening to patients’ stories and exploring options in a collaborative way will increase empathy and the ability to provide care in the best way [24, 31, 39] • Planning for death with individuals who have spent so much energy surviving requires an understanding of their life experiences [39] *Flexible programmes and availability* • A mobile and flexible program and being available through multiple modalities (e-mail, phone etc.) improves communication [45] • Provide access to a range of activities to tackle the issue of boredom [34]  *Advance care planning* • Every patient at the time of hospital admission should be asked who their surrogate decision-maker is and how to contact that person [40] • Primary care physicians should address preferences regarding end-of-life care with homeless patients in the primary setting, and (whenever possible) a primary care physician should be called to help hospital physicians make such decisions [40] • Addressing themes concerning dignity and asking questions about death and dying in advance directive formats [31] *Support after death* • Identification of proxies not only for medical decision-making (as traditionally identified) but also for witnessing and care of the body after death [33] • Support after death for both shelter occupants and staff, including bereavement groups and memorial services from friends and family [45] | *Availability of accommodation* • Ensure that palliative care beds are accessible to people who are homeless, e.g. by a palliative care team that is able to make use of limited space, operate in many different locations and provide flexible treatment options or special accommodation to facilitate dying for homeless people [34, 45] • Provision of appropriate accommodation with healthcare support for homeless individuals who are too sick for hostels and not sick enough for hospital [34]  *Involved expertise and coordination* • Having a Palliative Care Coordinator [32, 34] • A palliative care team that supports shelter staff and is able to make use of limited space, operate in many different locations and provide flexible treatment options [45]  *Policies and guidelines*  • Hospital discharge policies and establishment of a pathways model [34] • Guidelines of communication regarding communication on death and dying need reconsideration because communication needs to be sensitively weighted against the risk of harming the patient by exposing the social loneliness and intensifying feelings of anxiety and fear about the impending death [37] • Institutions should consider developing an explicit policy for involving additional persons, such as hospital ethics committees and/or advocates for homeless persons [40] *Partnering and exchange of knowledge between organizations* • Partnering social communities with the end-of-life care system. Trust developed between agencies and homeless populations can help mediate access to a range of other services and can provide accurate and up-to-date medical information and clearer lines of communication. Partnerships need to be developed [25, 27, 35] • The community palliative care service should initiate discussions with hospital referrers and community nurses about ways to approach referrals and shared care of terminally ill homeless people, as well as about how best to provide information on the range of services available to homeless people [47] • There must be diversity in institutions and palliative services (hospital-based palliative care, shelter-based palliative care, street-based palliative care. Individuals have different levels of comfort [36] • The population needs to advocate the importance of palliative care in-shelter [45] • Primary and continuous care are possible for elderly homeless people if multidisciplinary teams of doctors working with nurse practitioners, physician assistants, nurses, social workers and benefits specialists based in hospitals and health centres are willing to venture out on the streets on a consistent basis to offer services directly [44] |
